# Whole-Body **^18^**FDG-PET in an Arthritis Paraneoplastic Syndrome Revealed an Underlying Hematological Neoplasm

**DOI:** 10.1155/2013/594704

**Published:** 2013-06-02

**Authors:** Serge Cammilleri, Marie Caroline Guzian, Jean-Pierre Mattei, Olivier Mundler, Sandrine Guis

**Affiliations:** ^1^Service de Médecine Nucléaire, AP-HM, Faculté de Médecine de Marseille, Hôpital La Timone, Aix-Marseille University, 27 Boulevard Jean Moulin, 13005 Marseille, France; ^2^Service de Rhumatologie, AP-HM, Faculté de Médecine de Marseille, Hôpital Sainte Marguerite, Aix-Marseille University, 27 Boulevard Jean Moulin, 13005 Marseille, France; ^3^Service de Rhumatologie 1, Hôpital de Sainte Marguerite, 270 Bd de Sainte Marguerite, 13009 Marseille, France

## Abstract

We showed the first image of ^18^FDG-PET, which leads to a diagnosis of lymphoma in an atypical polyarthritis. About 4% of patients with lymphoma or leukemia suffered from rheumatologic paraneoplastic symptoms like arthralgia and about 10% of the patients with rheumatologic or neurologic clinical symptoms develop a solid cancer or hematological neoplasm. ^18^FDG-PET is an interesting exam to identify an underlying malignancy when a paraneoplastic syndrome is suspected; it can detect the primitive lesion and/or the metastasis lesions. The use of the ^18^FDG-PET can help to detect earlier hematological neoplasm in cases of paraneoplastic syndrome without a determined cause and to treat more rapidly and specifically the patient.

In rheumatology, rare cases of nonspecific inflammatory rheumatisms with arthralgias and arthritis are paraneoplastic syndrome. About 10% of the patients with rheumatologic or neurologic clinical symptoms develop a solid cancer or hematological neoplasm [1–3]. Exploration with [18F]-fluorodeoxyglucose (^18^FDG) positron emission tomography (PET) was used for diagnosis, the staging notably with the detection of bone metastasis (sensitivity from 62% to 100% depending on the primitive tumor and specificity from 96% to 100%), and the monitoring of the treatment of malignant pathologies and hematological neoplasms are recognized and essential. In fact, ^18^FDG-PET is a molecular and metabolic imaging modality which combines the metabolic data of PET with morphological data from computed tomography CT. ^18^FDG-PET is an interesting exam to identify an underlying malignancy when a paraneoplastic syndrome is suspected, and it can detect the primitive lesion and/or the metastasis lesions [4].

We present ^18^FDG-PET result of a 73-year-old man with seronegative symmetric inflammatory polyarthritis. He had a history of wrist, metacarpophalangeal, and ankle and knee joints arthralgias for two years which increased for 10 months without an inflammatory biological syndrome (ESR: 5 mm at first hour and PCR: 5 mg/L). He was hospitalized because the arthritis of the ankles and knees appeared with significantly swollen and warm joints. The stiffness increased in the morning and lasted for two hours. He had no fever or fatigue but a progressive loss of 6 kilos in the last year and a half. Erythrocyte sedimentation rate was 5 mm in the first hour, C reactive protein was 5 mg/L, and blood count was normal. Rheumatoid factors, ACPA, and antinuclear antibodies were negative. Body computed tomography (CT) did not show specific paratracheal infracentimetric lymph node without tissular lesion. The ^18^FDG-PET result (Figures 1(a), 1(b), 1(c), and 1(d)) showed the presence of  hypermetabolical heterogenous seats in the bone marrow in the bones of the upper and lower limbs especially in the lesser trochanters (maximal standard uptake value (msuv) = 6.1) and also the spleen (msuv = 2.9 versus msuv liver 2.3, ratio 1.2). So the images evoked a diagnosis of hematological neoplasm. The bone marrow biopsy and the myelogram done after the results of the ^18^FDG-PET gave the diagnosis of a follicular B lymphoma with a partial infiltration of 21% of lymphoid cells [5]. An R-CHOP (rituximab, cyclophosphamide, doxorubicine, vincristine, and prednisone) protocol treatment began.

About 4% of patients with lymphoma or leukemia suffered from rheumatologic paraneoplastic symptoms like arthralgias. Symptoms like polyarthritis are rare and no clinical or biological signs are specific. The paraneoplastic polyarthritis is most often asymmetric and when it is symmetric, a rheumatoid polyarthritis is mimed with clinical inflammatory signs of the wrist and the hands but without bone structural lesions and rheumatoid factors or ACPA detection. More often, the age of the patient is more than 50. The delay between the first rheumatological signs and the hematological neoplasm or solid malignancy varied between some weeks and some months. In 1995, Naschitz et al. reported that 23% of patients with initial unclassed inflammatory rheumatism and without signs of a tumor had a malignant lesion two years after the beginning of rheumatologic symptoms [1–3, 6]. The ^18^FDG-PET after diagnosis could be used to detect 15%–20% of unknown lesions and to monitor the treatment and followup [7–9]. Moreover, this exam allows us not only to visualize the extramedullar lesions but also to point out the medullar activity, which is a more serious prognosis of the disease [10]. The medullar activity is correlated to the tumoral proliferation [5, 11]. After a review of the literature, only one reported case of a hematological neoplasm was detected after ^18^FDG-PET, and it was a young woman with bone inflammatory pain and with severe inflammatory biological syndrome. The case was different from ours where a diagnosis of acute leukemia was given thanks to a ^18^FDG-PET exam [2].

 We showed in this observation the first image of  ^18^FDG-PET, which leads to a diagnosis of lymphoma in an atypical polyarthritis. The ^18^FDG-PET is one of the important paraclinical exams in rheumatology (for neoplastic disease but also new developments in inflammatory diseases are described). In our case, it was a very useful exam because it pointed out hypermetabolic lesions and their sites and evoked diagnosis. ^18^FDG-PET can lead clinicians towards a diagnosis. The use of the ^18^FDG-PET can help to detect earlier hematological neoplasm in cases of paraneoplastic syndrome without a determined cause and to treat more rapidly and specifically the patient.

## Figures and Tables

**Figure 1:**
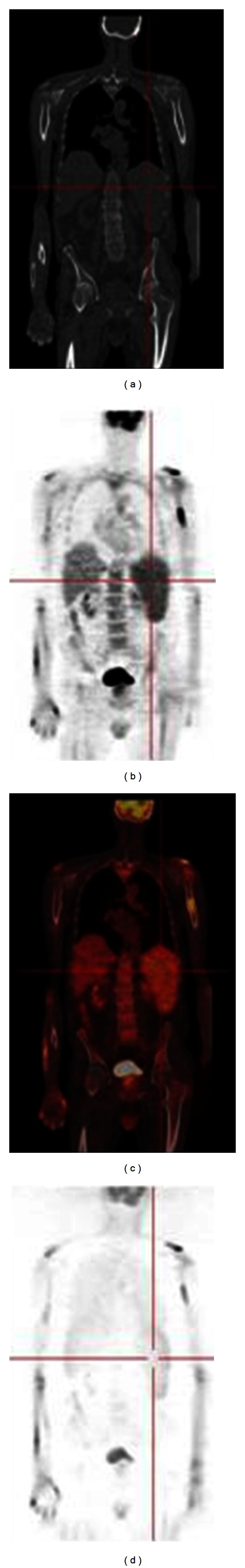
The whole-body  ^18^FDG-PET exam. Selected coronal slices of computed tomographic scan (a); F-18 FDG PET scan (b); PET-CT fusion (c); maximum intensity projections (d). Intense heterogeneous activity maximal standard uptake value (msuv) = 6.1 on bone marrow and splenic tissue msuv = 2.9 (b).
